# Occupational stress is associated with major long-term weight gain in a Swedish population-based cohort

**DOI:** 10.1007/s00420-018-1392-6

**Published:** 2018-12-06

**Authors:** Sofia Klingberg, Kirsten Mehlig, Ingegerd Johansson, Bernt Lindahl, Anna Winkvist, Lauren Lissner

**Affiliations:** 1 Section for Epidemiology and Social Medicine (EPSO), Department of Public Health and Community Medicine, Sahlgrenska Academy, University of Gothenburg, P.O. Box 453, 405 30 Gothenburg, Sweden; 2 Department of Odontology, Umeå University, Umeå, Sweden; 3 Department of Public Health and Clinical Medicine, Nutrition Research, Umeå University, Umeå, Sweden; 4 Department of Public Health and Clinical Medicine, Occupational and Environmental Medicine, Umeå University, Umeå, Sweden; 5 Department of Internal Medicine and Clinical Nutrition, Sahlgrenska Academy, University of Gothenburg, Gothenburg, Sweden

**Keywords:** Job strain, Work stress, Job demand, Decision latitude, Weight gain, Weight change, Prospective study

## Abstract

**Purpose:**

Occupational stress and obesity are both increasing in prevalence, but prospective findings relating these conditions are inconsistent. We investigated if baseline as well as prolonged exposure to high job demands and low decision latitude were associated with major weight gain (≥ 10% of baseline weight) in 3872 Swedish women and men examined three times over 20 years in the population-based Västerbotten Intervention Program.

**Methods:**

Anthropometry was measured and participants completed questionnaires on job strain, diet, and other lifestyle factors. Logistic regression was used to estimate odds ratios (OR) and 95% confidence intervals (CI), adjusting for confounders.

**Results:**

Adjusting for age, baseline low decision latitude was associated with major weight gain over 10- and 20-year OR (95% CI) 1.16 (1.00–1.33) and 1.29 (1.13–1.47), respectively (both sexes combined). After adjustment for diet quality and other confounders, the effect over 20 years remained 1.30 (1.13–1.50). Sex modified the effect of prolonged exposure to high job demands over at least 10 years (interaction *p* = 0.02), showing that high job demands was a risk factor of major weight gain over 20 years in women [1.54 (1.14–2.07)], but not in men [0.87 (0.63–1.19)]. Neither diet nor other lifestyle factors explained these associations.

**Conclusions:**

In conclusion, low decision latitude predicted major weight gain in women and men. In women, the results suggest an additional contribution to major weight gain from high job demands.

**Electronic supplementary material:**

The online version of this article (10.1007/s00420-018-1392-6) contains supplementary material, which is available to authorized users.

## Introduction

The population mean body mass index (BMI) has increased in recent decades and a prognosis forecasts future increases in obesity prevalence (Breda et al. 2015). While a positive energy balance, originating from excess energy intake relative to energy expenditure, is a fundamental cause for weight gain, psychosocial factors such as mental stress might be contributing factors. Effects of mental stress on weight gain could be mediated through unhealthy behaviors such as low diet quality. In addition, chronic stress could, through higher cortisol levels, impact on visceral fat accumulation and reduction in lean body mass (Kyrou and Tsigos 2009).

Alongside the trend of increasing BMI, the prevalence of self-reported mental stress has also increased (Lissner et al. 2008). Work-related stress, also referred to as job strain, with high demands and low decision latitude as conceptualized by Karasek and Theorell (1990), has also been reported to be higher in later years (Malard et al. 2015; Utzet et al. 2015) as have the prevalence of other work-related stress exposures (Houdmont et al. 2012). Although changing social norms regarding the stress concept may explain some part of the increased prevalence of self-reported stress, a considerable increase in sickness absence due to psychiatric diagnoses, including the fatigue syndrome, during the last decade has been reported in Sweden, providing support for a factual increase in stress (Swedish Social Insurance Agency 2016).

Job strain has been shown to predict obesity-related diseases such as diabetes (Nyberg et al. 2014), coronary heart disease (Kivimäki et al. 2012), and stroke (Fransson et al. 2015). However, the prospective association between job strain and weight gain, as recently compiled in a systematic review and meta-analysis (Kivimäki et al. 2015), is not consistent and does overall not support the hypothesis that job strain promote weight gain. However, two of the four included studies on job strain in relation with weight gain used self-reported weight, and only one of them was truly population-based, questioning both validity in the outcome and generalizability of the results. In this study, we report on the association between exposure to high job demands and low decision latitude as well as job strain, and major weight gain over 10 and 20 years in a population based cohort of Swedish women and men, aged 30 or 40 years at baseline, with additional focus on the concurrent influence of diet quality.

## Methods

### Study population

The Västerbotten Intervention Program (VIP) is an ongoing population-based study of adults in northern Sweden (http://www.biobank.umu.se/nshds/). The project started in 1985 and invites inhabitants of Västerbotten County to a health examination the year they turn 40, 50, and 60 years of age. Until 1995, those turning 30 were also invited. Thus, inhabitants in Västerbotten County have been able to participate in VIP on multiple occasions. Between 1991 and 1995 participation rates varied between 48 and 57%, but after the decision to omit those turning 30 years, participation rates have increased to 66–67% since 2005 (Norberg et al. 2010). An analysis of the drop out in 1992 and 1993 showed small differences between participants and non-participants in that non-participants were somewhat more likely to be unemployed, to have lower income, and to be younger (Weinehall et al. 1998). In this study, we use measured data on height and weight as well as questionnaire data on work-related stress, diet, and other lifestyle factors as described below. Data were collected at baseline, 10- and 20-year follow-up for all participants included in this study (study sample described below).

### Anthropometry

Height was measured standing without shoes to the nearest cm. Weight was measured in light clothing to the nearest kg. Body mass index (BMI) was calculated by dividing weight (kg) by height squared (m^2^). Major weight gain was defined as an increase in body weight of ≥ 10% of baseline body weight at either follow-up examination (10 and 20 years). This cutoff was used based on the suggestion by Stevens et al. that long-term weight maintenance in adults could be defined as a weight change of less than 3% body weight and that weight changes of between 3 and 5% should be considered small weight fluctuations (Stevens et al. 2006).

### Work stress

Work stress, also referred to as job strain, was measured according to a Swedish modification of the questionnaire developed by Karasek et al. (1998). Psychological job demands were measured using four items, and decision latitude, comprising skill discretion and decision authority, was measured using six items (Supplemental file 1). Each item had four response alternatives forming a four-point ordinal scale. An index of psychological job demands was formed by the sum of the four items, with four as the minimum and 16 as the maximum score. Correspondingly, an index of decision latitude was formed by the sum of the six items, with six as the minimum and 24 as the maximum score. Psychological job demands and decision latitude were both dichotomized into “low” and “high” by the gender specific median score with the median included in the lower category (10 for psychological job demands for both sexes and 18 and 19 for decision latitude, in women and men, respectively). Cross classification of psychological job demands and decision latitude formed the job strain quadrant model with four distinct groups of job strain: “low strain” (low psychological demands and high decision latitude), “active” (high psychological demands and high decision latitude), “passive” (low psychological demands and low decision latitude), and “high strain” (high psychological demands and low decision latitude). Prolonged exposure to occupational stress was defined in a sub-group classified in the same job strain category at both baseline and at 10-year follow-up. Social support at work was assessed by four questions and likewise dichotomized by the gender specific median score (9 for women and 8 for men).

### Dietary intake

Habitual diet during the last year was assessed at baseline by a food frequency questionnaire (FFQ) with 84 questions. The FFQ was validated with 10 repeated 24-h recalls and concluded to have validity comparable to other FFQs used in other cohort studies (Johansson et al. 2002; Klingberg et al. 2013). In addition, the FFQ has been validated by serum biomarkers (Johansson et al. 2010; Wennberg et al. 2009). Intake frequency for each item was given on a nine-level scale varying from never to four or more times a day. Portion sizes for staple foods, meat/fish, and vegetables, respectively, were indicated by four color photos representing different portions. For other foods, sex- and age-specific portions determined from the validation study (Johansson et al. 2002) or standard portions (National Food Agency Sweden 1999) were used.

A healthy diet index, developed by Drake et al. (2011), based on the Swedish Nutrition Recommendations and dietary guidelines, was calculated for each participant. This index was based on adherence to the following criteria: sucrose ≤ 10% of energy intake (*E*%); saturated fat ≤ 13 *E*%; polyunsaturated fat between 5 and 10 *E*%; dietary fibre ≥ 2.4 g/MJ; fruits and vegetables ≥ 400 g/day; and fish ≥ 300 g/week. One point was given for each criteria met. Thus, the index ranged from zero to six points with a higher point indicating a higher diet quality. The total index was then categorized as low (≤ 1 point), intermediate (2–3 points), and high diet quality (≥ 4 points). Only 2.9% of the population complied with the recommendation of maximum 10 *E*% saturated fat, and therefore, the cutoff was set at 13 *E*% after the addition of one standard deviation of the mean population intake. As the recommendation of dietary fibre intake per energy unit has no defined lower limit, we set the lower limit of fibre intake at 2.4 g/MJ after subtraction of one standard deviation of the mean population intake from the recommendation of approximately 3 g/MJ. As the intake of fruits and vegetables did not include juice, the cutoff was set at 400 g/day instead of 500 g/day.

### Lifestyle and other variables

Physical activity was defined by the validated Cambridge Index of Physical Activity (InterAct Consortium 2012) which is based on two questions, one regarding physical activity at work and one regarding leisure time physical activity. The index classifies each participant as either inactive, moderately inactive, moderately active or active. Smoking was categorized as current smoker or ex-smoker/never smoker, respectively. Level of education was derived from a question with four response alternatives and coded into a binary variable of academic education (yes/no).

### Study sample

**Fig. 1:**
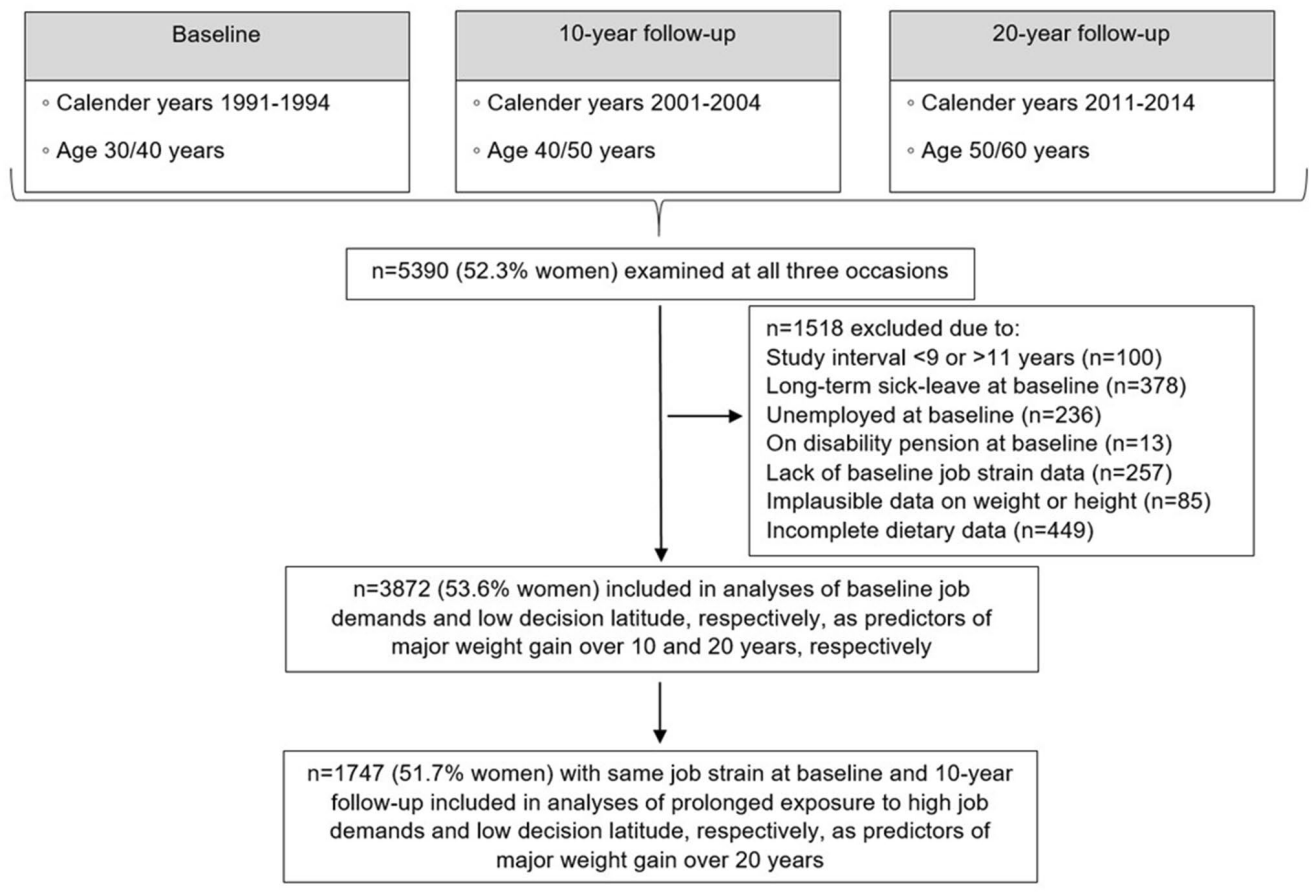
Overview of study design and study flowchart for the current analyses

Data for this study were eligible from 1991 and onwards. From 1991 until 2014 5390 individuals (52.3% women) participated in health examinations at three occasions over a period of approximately 20 years (Fig. 1). Participants who were examined at intervals shorter than 9 years or longer than 11 years between baseline visit and the first follow-up or between the first and the second follow-ups, respectively, were excluded from the analyses (*n* = 100). Similarly, participants who were on long term (> 6 months) sick leave at baseline (*n* = 378), unemployed (*n* = 236) or on disability pension (*n* = 13), or lacked baseline data on one or more items of the job demand and decision latitude scale (*n* = 257) were excluded. Participants with missing or implausible data on weight (< 30 kg) or height (< 130 cm) (*n* = 85) at any of the examinations were excluded. Additionally, participants were excluded if they had incomplete dietary data (*n* = 449) including missing responses to more than 10% of the FFQ questions or one or more missing portion size response, as described elsewhere (Johansson et al. 2002). Hence, the main analytical sample consisted of 3872 individuals, of which 2074 were women and 1798 were men. A total of 1747 participants (904 women and 843 men), who also had complete data on job demand and decision latitude at the 10-year follow-up and were classified within the same job strain category at both baseline and 10-year follow-up were selected for analyzes of the association between prolonged exposure to occupational stress and major weight gain over 20 years. All statistical analyses were performed for both sexes combined and separately for women and men.

### Statistics

Logistic regression was used to assess the association between major weight gain and exposure to high job demands and low decision latitude (mutually adjusted for each other). The results are presented as odds ratios (OR) with 95% confidence intervals (CI). Model 1 was adjusted for baseline age and sex (when sexes were analysed together). Model 2 was further adjusted for baseline diet quality, social job support, physical activity, smoking, marital status, academic education, and BMI. The following interactions were tested by inclusion of the corresponding product terms in addition to the main effects: sex by job demands; sex by decision latitude; job demands by decision latitude; and the respective main exposure variable (i.e., job demands and decision latitude) by the following covariates: age group; BMI category (< 25 kg/m^2^ or ≥ 25 kg/m^2^); academic education; and job support. Sensitivity analyses were performed after exclusion of participants with weight loss ≥ 5% of baseline body weight and after inclusion of participants with incomplete diet data. Furthermore, sensitivity analyses included baseline job demands and decision latitude as continuous variables. All statistical analyses were performed in SAS, version 9.3 (SAS institute, Cary, NC, USA). The significance level was set at 0.05 (two-sided tests).

## Results

Baseline characteristics of the study population are shown in Table 1. At baseline, mean BMI was below 25 kg/m^2^ in both sexes, but 27% of the women and 39% of the men were overweight or obese. Over the first 10 years, 33.5% of the women and 26.0% of the men gained ≥ 10% body weight, while over 20 years, these figures were 48.9% and 43.7% for women and men, respectively. The mean (sd) weight gain over the first 10 years was 4.6 (6.1) kg for women and 5.1 (6.8) kg for men, while over 20 years, these figures were 6.8 (8.1) kg and 7.1 (7.5) kg, respectively.

**Table 1:** Baseline characteristics of the VIP study sample

	Women and men (*n* = 3872)	Women (*n* = 2074)	Men (*n* = 1798)
General characteristics			
Aged 29–31 (%)	36.9	36.5	37.4
Aged 39–41 (%)	63.1	63.5	62.6
Academic education (%)	26.3	31.1	20.7
Married/cohabitant (%)	86.2	88.5	83.5
Current smoker (%)	24.3	26.6	21.7
Physically inactive^a^ (%)	47.7	48.3	47.0
Anthropometry			
Height (cm), mean (SD)	171.9 (9.3)	165.4 (5.8)	179.5 (6.4)
Weight (kg), mean (SD)	71.5 (12.6)	64.7 (9.9)	79.3 (10.6)
BMI (kg/m^2^), mean (SD)	24.1 (3.3)	23.7 (3.4)	24.6 (2.9)
BMI ≥ 25.0 kg/m^2^ (%)	32.7	27.0	39.3
Diet			
Energy intake (MJ), mean (SD)	8.2 (2.7)	7.3 (2.2)	9.2 (3.0)
Sugar (*E*%), mean (SD)	7.3 (2.9)	7.7 (2.8)	7.0 (3.0)
Saturated fat (*E*%), mean (SD)	14.9 (3.1)	14.3 (2.8)	15.6 (3.2)
Polyunsaturated fat (*E*%), mean (SD)	5.2 (1.6)	4.9 (1.5)	5.4 (1.6)
Dietary fibre (g/MJ), mean (SD)	2.4 (0.6)	2.6 (0.7)	2.2 (0.5)
Fruits and vegetables (g/day), mean (SD)	226 (166)	269 (175)	177 (140)
Fish (g/week), mean (SD)	291 (255)	327 (271)	250 (228)
Diet quality^b^, mean (SD)	2.5 (1.3)	2.7 (1.4)	2.3 (1.2)

^a^Inactive or moderately inactive

^b^Adherence to six criteria. One point for each criteria met; thus, a higher score indicates higher diet quality

Age-adjusted analyses in both sexes combined, showed low decision latitude to predict major weight gain over both 10 and 20 years (Table 2), while high job demands were not associated with the outcome. After adjustment for diet quality and other confounders, the effect of low decision latitude remained for major weight gain over 20 years. Interactions between sex and exposure were non-significant (data not shown). Analyses stratified by sex can be found in supplemental Table S1. Baseline diet was itself only associated with major weight gain over 10 years, with an increased risk of major weight gain in women and men reporting a high diet quality [OR (95% CI) 1.26 (1.06–1.50)] as compared to those reporting an intermediate diet quality. When participants with baseline BMI ≥ 25 kg/m^2^ were excluded, high diet quality was no longer associated with risk of major weight gain [OR (95% CI) 1.13 (0.91–1.39)].

**Table 2:** Odds ratio and 95% CI for the association between job demands and decision latitude (mutually adjusted) and weight gain ≥ 10% of baseline body weight over 10 and 20 years in 3872 women and men participating in VIP

Exposures	Outcome: ≥ 10% weight gain		Covariate adjustment for baseline	
	Follow-up time (years)	Cases (%)	Age group and sex	+ Diet quality, social job support, physical activity, smoking, marital status, academic education and BMI
High job demands^a^	10	1161 (30.0)	1.07 (0.93–1.24)	1.14 (0.98–1.32)
Low decision latitude^b^			1.16 (1.00–1.33)*	1.12 (0.96–1.31)
High job demands^a^	20	1799 (46.5)	1.06 (0.93–1.21)	1.05 (0.92–1.21)
Low decision latitude^b^			1.29 (1.13–1.47)***	1.30 (1.13–1.50)***

*p < 0.05, **p < 0.01, ***p < 0.001

^a^Reference category low job demands

^b^Reference category high decision latitude

To study prolonged exposure, 1747 participants (904 women and 843 men) were identified who were classified in the same job strain quadrant category at baseline and at 10-year follow-up, representing 45% of the study sample. Prolonged exposure to low decision latitude predicted major weight gain when women and men were analyzed together (Table 3), while no association was seen for high job demands. However, a significant interaction between sex and job demands was found (*p* = 0.02). Analyses stratified by sex showed that prolonged exposure to high demands was associated with an increased risk of major weight gain over 20 years in women, while high job demands did not predict major weight gain in men. A significant interaction was found in women between prolonged exposure to high demands and baseline BMI category (*p* = 0.01). Stratification by baseline weight category showed that prolonged exposure to high demands was detrimental only in women with baseline overweight [OR (95% CI) 3.13 (1.67–5.86) vs. 1.24 (0.85–1.79) in women with normal weight at baseline].

**Table 3:** Odds ratio and 95% CI for the association between prolonged exposure to high job demands and low decision latitude (mutually adjusted) and weight gain ≥ 10% of baseline weight over 20 years in 1754 women and men participating in VIP

	Prolonged exposure i.e. same exposure at baseline and 10 years follow up	Outcome: ≥ 10% weight gain	Covariate adjustment for baseline	
		Cases (%)	Age group and sex	+ Diet quality, job support, physical activity, smoking, marital status, academic education and BMI
Women and men (*n* = 1747)	High job demands^a^	789 (45.2)	1.15 (0.94–1.40)	1.16 (0.94–1.44)^c^
	Low decision latitude^b^		1.33 (1.09–1.63)*	1.32 (1.05–1.67)*
Women (*n* = 904)	High job demands^a^	424 (46.9)	1.43 (1.08–1.88)*	1.54 (1.14–2.07)**
	Low decision latitude^b^		1.23 (0.94–1.62)	1.16 (0.84–1.62)
Men (*n* = 843)	High job demands^a^	365 (43.3)	0.91 (0.67–1.22)	0.87 (0.63–1.19)
	Low decision latitude^b^		1.45 (1.08–1.95)*	1.44 (1.03–2.01)*

*p < 0.05, **p < 0.01, ***p < 0.001

^a^Reference category low job demands

^b^Reference category high decision latitude

^c^Significant interaction between job demand and sex *p* = 0.02

Interactions between job demand and decision latitude were neither significant for baseline exposure nor for prolonged exposure. Results from analyses investigating exposure according to the job strain quadrant model are presented in supplemental Table S2 (baseline exposure) and Table S3 (prolonged exposure) and show that the high strain group was most vulnerable to major weight gain.

Sensitivity analyses (data not shown) of both baseline occupational stress and prolonged occupational stress showed that neither excluding subject who lost ≥ 5% of baseline body weight over 10 (*n* = 202) and 20 years (*n* = 224), respectively, nor including participants with incomplete diet data (*n* = 449) affected the results. Treating baseline exposures as continuous variables did not change the conclusions (data not shown).

## Discussion

In this study, we observed that low decision latitude independently and consistently predicts long-term major weight gain in a cohort of Swedish women and men. Moreover, considering prolonged exposure the present study provided suggestive results of a sex-specific effect of high job demands in women, which was more pronounced in women with overweight at baseline.

As far as we know, there is only one previous study investigating associations between the demand-control model and weight gain with comparable follow-up time. That study demonstrated a dose–response association between prolonged iso-strain, defined as high strain in combination with low social support, and incident obesity over 19 years in women and men (Brunner et al. 2007). Furthermore, for the three dimensions of iso-strain—high job demands; low decision latitude; and low social support—significant associations were only found for prolonged low social support at work in men (Brunner et al. 2007). Women did exhibit elevated, although non-significant, odds of obesity for all three dimensions (Brunner et al. 2007). Another study (Niskanen et al. 2017) investigating changes in working conditions in relation with weight gain (≥ 10%) reported that prolonged exposure to high job demands was associated with major weight gain over 12 years in women but not in men, while no association was seen for prolonged exposure to low decision latitude in women or in men. Other previous studies with shorter follow up of between 2 and 7 years have not found evidence of an association between baseline job strain and weight gain or risk of obesity in either sex (Ishizaki et al. 2008; Nyberg et al. 2012), or only found associations in women (Kivimäki et al. 2006; Roos et al. 2013; Shields 1999). In models adjusted for several covariates, Shields (1999) found females with high baseline job strain to have increased odds of a weight gain of more than 8.8% of baseline body weight over 2–3 years, while Roos et al. (2013) showed that females belonging to the passive group had an increased odds of gaining 5 kg or more over 5–7 years. Furthermore, Kivimäki (2006) found a positive association between job demands and weight gain in women. Comparability of study results is problematic because of discrepancies in both exposure and outcome assessment. Moreover, although most previous studies reported analyses stratified by gender, support for gender specific effects of job strain in relation with weight gain was inconsistent. However, in accordance with results reported by Niskanen et al. (2017) and supported by a significant interaction between sex and high job demands, we found prolonged exposure to high job demands to stand out as detrimental only in women. One plausible explanation is that a female sensitivity to prolonged high job demands could relate to women’s double work load due to higher responsibility for domestic work, which appears also in a relatively gender-equal society as Sweden (Harryson et al. 2012).

The link between job strain and weight gain could potentially work through, for example, dietary intake, physical activity, or metabolic rate, but neither physical activity nor diet quality explained the associations seen in the present study. The diet quality index used in the present study, which was developed in another Swedish cohort (Drake et al. 2011) has previously been shown to inversely associate with total mortality as well as cardiovascular disease morbidity and mortality (Drake et al. 2013; Hlebowicz et al. 2013), while no association was found for diabetes risk (Mandalazi et al. 2016). The diet index has to our knowledge not previously been used in prediction of weight gain, and it is plausible that such an index is not optimal for that purpose, due to obesity-related reporting biases. Because neither changes in metabolic rate, nor changes in body composition or regional fat pattering were measured in the VIP cohort, we can only speculate in that exposure to job strain, with potential exposure to higher levels of the catabolic hormone cortisol, could have had detrimental effects on muscle mass with lower basal metabolic rate and weight gain as a consequence. The primary strength of this study is the repeated assessment of both exposure and outcome with the ability to investigate associations between prolonged exposure to high demands and low decision latitude, respectively, and weight gain over one and two decades. The examination interval of 10 years was not explicitly chosen for the present study, but was likely relevant for our outcome of interest, since many previous studies with shorter study interval have failed to show significant associations between job strain and major weight gain. The use of validated instruments to assess exposure to both job strain and other lifestyle factors, and standardized measurement of outcome performed by medical staff also deserve to be highlighted as important strengths. The outcome was defined as a relative gain in baseline weight of 10% or more, and an alternative would have been to use an absolute cut-off for weight gain instead such as that of 5 kg or more as suggested by World Health Organization (2003). The use of a relative weight change cutoff is, however, supported by increased congruence across body sizes (Stevens et al. 2006).

This study has potential limitations that need to be considered. First, it has to be acknowledged that the exposure to the two dimensions of job strain is self-reported by participants and may introduce various forms of bias to the analysis and results. Possible fluctuations in exposure during follow-up must also be acknowledged, although such fluctuations would be likely to attenuate our observed effect estimates. Second, although the full VIP cohort is population based with an essentially representative participation (Weinehall et al. 1998), the current VIP sub-sample that attended three subsequent examinations, may be subject to participation bias. However, we recently showed that a sub-sample of the VIP cohort with two repeated measures (Winkvist et al. 2017) did not exhibit any essential socio-demographic differences compared to the full VIP cohort. Third, we were only able to use baseline data for dietary intake, since the assessment method was changed from an 84 item FFQ to a 64 item FFQ at the follow-ups. Finally, the present study lacked data on metabolic rate, body composition, and regional fat patterning.

In conclusion, the present study supports the hypothesis that occupational stress promotes weight gain. Neither baseline diet quality, physical inactivity, nor other covariates, explained the results, therefore, pointing at other mechanisms underlying these associations. To further understand the mechanisms between job strain and weight gain, studies on changes in both regional fat patterning and body composition in relation with job strain are warranted. Although the association between occupational stress and major weight gain seems rather weak, the public health relevance of our findings could be important because of the increasing prevalence of work-related stress. The importance of these findings is likely to go beyond the risk of unhealthy weight gain, since the identification of vulnerable groups and gender-sensitive preventive efforts to reduce job strain have the potential to not only reduce weight gain, but also risk of cardiovascular disease and diabetes.

## Electronic supplementary material

Below is the link to the electronic supplementary material.

Supplementary material 1 (DOCX 22 KB)

Supplementary material 2 (DOCX 12 KB)
