# Supplementary material for: Occupational stress is associated with major long-term weight gain in a Swedish population-based cohort
Source: Int Arch Occup Environ Health. 2018 Dec 6;92(4):569–76. doi: 10.1007/s00420-018-1392-6 (PMC6435615; doi:10.1007/s00420-018-1392-6)
Supplement: Supplementary file 1 — Supplementary material 1 (DOCX 22 KB) [file 420_2018_1392_MOESM1_ESM.docx]

Supplementary table S1. Odds ratio and 95% CI for the association between job demands and decision latititude (mutually adjusted) and weight gain ≥10% of baseline body weight over 10 and 20 years in 2074 women and 1798 men participating in VIP.

|  | Exposures | Outcome: ≥10% weight gain | | Covariate adjustment for baseline | |
| --- | --- | --- | --- | --- | --- |
|  |  | Follow-up time | Cases (%) | age group and sex | + diet quality, social job support, physical activity, smoking, marital status, academic education and BMI |
|  |  |  |  |  |  |
| Women  (n = 2074) | High job demands^1^ | 10 years | 694 (33.5) | 1.21 (1.00-1.46) | 1.28 (1.05-1.56)* |
|  | Low decision latitude^2^ |  |  | 1.28 (1.06-1.54)** | 1.21 (0.98-1.50) |
|  | High job demands^1^ | 20 years | 1014 (48.9) | 1.13 (0.94-1.35) | 1.12 (0.93-1.36) |
|  | Low decision latitude^2^ |  |  | 1.23 (1.03-1.47)* | 1.26 (1.03-1.53)* |
| Men  (n = 1798) | High job demands^1^ | 10 years | 467 (26.0) | 0.92 (0.74-1.15) | 0.99 (0.79-1.25) |
|  | Low decision latitude^2^ |  |  | 1.00 (0.81-1.24) | 1.00 (0.79-1.26) |
|  | High job demands^1^ | 20 years | 785 (43.7) | 1.00 (0.82-1.22) | 1.02 (0.83-1.25) |
|  | Low decision latitude^2^ |  |  | 1.36 (1.12-1.65)** | 1.36 (1.10-1.68)** |

^1^ Reference category low job demands

^2^ Reference category high decision latitude

* p<0.05, ** p <0.01, *** p<0.001

Supplementary table S2. Odds ratio and 95% CI for the association between job strain and weight gain ≥10% of baseline body weight over 10 and 20 years in 3872 women and men participating in VIP.

|  | Job strain category | Follow-up time | Cases (%) | Covariate adjustment for baseline | |
| --- | --- | --- | --- | --- | --- |
|  |  |  |  | age group and sex | + diet quality, social job support, physical activity, smoking, marital status, academic education and BMI |
| Women and men (n=3872) | Low strain^1^ | 10 years | 259 (29.2) | 1 (ref) | 1 (ref) |
|  | Passive^2^ |  | 440 (30.1) | 1.05 (0.87-1.26) | 1.03 (0.85-1.26) |
|  | Active^3^ |  | 206 (27.1) | 0.94 (0.76-1.17) | 1.01 (0.80-1.26) |
|  | High strain^4^ |  | 256 (33.5) | 1.25 (1.01-1.54)* | 1.28 (1.03-1.60)* |
|  | Low strain^1^ | 20 years | 385 (43.5) | 1 (ref) | 1 (ref) |
|  | Passive^2^ |  | 706 (48.3) | 1.21 (1.02-1.44)* | 1.22 (1.02-1.46)* |
|  | Active^3^ |  | 319 (42.0) | 0.98 (0.80-1.20) | 0.97 (0.78-1.19) |
|  | High strain^4^ |  | 389 (50.9) | 1.37 (1.13-1.67)** | 1.38 (1.12-1.69)** |
| Women (n=2074) | Low strain^1^ | 10 years | 150 (29.4) | 1 (ref) | 1 (ref) |
|  | Passive^2^ |  | 267 (33.9) | 1.23 (0.96-1.56) | 1.17 (0.90-1.52) |
|  | Active^3^ |  | 122 (32.0) | 1.14 (0.85-1.52) | 1.22 (0.90-1.65) |
|  | High strain^4^ |  | 155 (39.3) | 1.55 (1.17-2.04)** | 1.56 (1.16-2.09)** |
|  | Low strain^1^ | 20 years | 232 (45.4) | 1 (ref) | 1 (ref) |
|  | Passive^2^ |  | 391 (49.6) | 1.18 (0.94-1.47) | 1.17 (0.92-1.50) |
|  | Active^3^ |  | 178 (46.7) | 1.06 (0.81-1.38) | 1.02 (0.77-1.35) |
|  | High strain^4^ |  | 213 (54.1) | 1.40 (1.07-1.82)* | 1.43 (1.08-1.89)* |
| Men (n=1798) | Low strain^1^ | 10 years | 103 (29.1) | 1 (ref) | 1 (ref) |
|  | Passive^2^ |  | 173 (25.6) | 0.83 (0.63-1.10) | 0.96 (0.66-1.41) |
|  | Active^3^ |  | 84 (22.2) | 0.72 (0.52-1.01) | 0.97 (0.63-1.49) |
|  | High strain^4^ |  | 101 (27.3) | 0.93 (0.68-1.28) | 1.09 (0.71-1.67) |
|  | Low strain^1^ | 20 years | 153 (40.8) | 1 (ref) | 1 (ref) |
|  | Passive^2^ |  | 315 (46.7) | 1.26 (0.97-1.63) | 1.28 (0.97-1.69) |
|  | Active^3^ |  | 141 (37.3) | 0.91 (0.68-1.22) | 0.94 (0.69-1.28) |
|  | High strain^4^ |  | 176 (47.6) | 1.36 (1.01-1.82)* | 1.38 (1.01-1.88)* |

* p<0.05, ** p <0.01, *** p<0.001

^1^ low demands, high decision latitude, ^2^ low demands, low decision latitude, ^3^ high demands, high decision latitude, ^4^ high demands, low decision latitude

Supplementary table S3. Odds ratio and 95% CI for the association between prolonged exposure to job strain and weight gain ≥10% of baseline body weight over 20 years in 1747 women and men participating in VIP.

|  | Prolonged exposure to job strain (i.e. same exposure at baseline and follow up) | Cases (%) | Covariate adjustment for baseline | |
| --- | --- | --- | --- | --- |
|  |  |  | age group and sex | + diet quality, social job support, physical activity, smoking, marital status, academic education and BMI |
| Women and men (n=1747) | Low strain^1^ | 133 (39.5) | 1 (ref) | 1 (ref) |
|  | Passive^2^ | 339 (47.4) | 1.33 (1.02-1.74)* | 1.33 (0.99-1.77) |
|  | Active^3^ | 170 (42.5) | 1.15 (0.86-1.55) | 1.16 (0.84-1.58) |
|  | High strain^4^ | 147 (50.0) | 1.51 (1.11-2.08)* | 1.53 (1.10-2.15)* |
| Women (n=904) | Low strain^1^ | 78 (40.0) | 1 (ref) | 1 (ref) |
|  | Passive^2^ | 164 (46.3) | 1.26 (0.88-1.80) | 1.17 (0.78-1.74) |
|  | Active^3^ | 101 (49.3) | 1.47 (0.99-2.19) | 1.54 (1.00-2.37)* |
|  | High strain^4^ | 81 (54.0) | 1.74 (1.13-2.69)* | 1.79 (1.12-2.85)* |
| Men (n=843) | Low strain^1^ | 55 (38.7) | 1 (ref) | 1 (ref) |
|  | Passive^2^ | 175 (48.3) | 1.42 (0.96-2.12) | 1.42 (0.92-2.19) |
|  | Active^3^ | 69 (35.4) | 0.88 (0.56-1.39) | 0.85 (0.53-1.38) |
|  | High strain^4^ | 66 (45.8) | 1.31 (0.82-2.11) | 1.25 (0.76-2.06) |

* p<0.05, ** p <0.01, *** p<0.001

^1^ low demands, high decision latitude, ^2^ low demands, low decision latitude, ^3^ high demands, high decision latitude, ^4^ high demands, low decision latitude
