# Supplementary material for: Occupational stress is associated with major long-term weight gain in a Swedish population-based cohort
Source: Int Arch Occup Environ Health. 2018 Dec 6;92(4):569–76. doi: 10.1007/s00420-018-1392-6 (PMC6435615; doi:10.1007/s00420-018-1392-6)
Supplement: Supplementary file 2 — Supplementary material 2 (DOCX 12 KB) [file 420_2018_1392_MOESM2_ESM.docx]

**Job demands (4 to 16 points – high score = high demands)**

**Does your work demand you to work very fast?**

Yes often=4, Yes sometimes=3, No seldom=2, No almost never=1

**Is your work mentally strenuous?**

Yes often=4, Yes sometimes=3, No seldom=2, No almost never=1

**Do you have enough time for your assignments?**

Yes often=1, Yes sometimes=2, No seldom=3, No almost never=4

**Does your work involve conflicting demands?**

Yes often=4, Yes sometimes=3, No seldom=2, No almost never=1

**Decision latitude (6 to 24 points – low score = low control)**

**Do you learn new things through your work?**

Yes often=4, Yes sometimes=3, No seldom=2, No almost never=1

**Does your work demand a high level of skill?**

Yes often=4, Yes sometimes=3, No seldom=2, No almost never=1

**Does your work require you to be inventive?**

Yes often=4, Yes sometimes=3, No seldom=2, No almost never=1

**Do you have to do the same things over and over again?**

Yes often=1, Yes sometimes=2, No seldom=3, No almost never=4

**Do you have a choice in deciding how you do your work?**

Yes often=4, Yes sometimes=3, No seldom=2, No almost never=1

**Do you have a choice in deciding what you do?**

Yes often=4, Yes sometimes=3, No seldom=2, No almost never=1
